# Bibliometric analysis of mucosal immunity in IgA nephropathy from 1990 to 2022

**DOI:** 10.1002/iid3.1156

**Published:** 2024-01-24

**Authors:** Xian Chen, Zhe Yan, Qing Pan, Chunxia Zhang, Yakun Chen, Xuzhi Liang, Shaomei Li, Gang Wu

**Affiliations:** ^1^ Department of Nephrology The Second Hospital of Hebei Medical University Shijiazhuang China; ^2^ Renal Division, The Affiliated Suzhou Hospital of Nanjing Medical University Suzhou Municipal Hospital Suzhou China

**Keywords:** bibliometric analysis, CiteSpace, gut microbiota, IgA nephropathy, mucosal immunity

## Abstract

**Objective:**

The study aimed to conduct a bibliometric analysis of mucosal immunity in IgA nephropathy (IgAN) and indicate its current status, hot sopts, and direction of future studies.

**Methods:**

The literature data was collected from the Web of Science Core Collection. CiteSpace 6.1.R3 was employed to conduct a visualization bibliometric analysis of mucosal immunity in IgA nephropathy, including authors, countries, journals, keywords, organizations, references, the bursts of keywords and references, and the timeline of keyword clusters and reference clusters.

**Results:**

A total of 315 publications from 1990 to 2022 were included. The number of articles in this field has increased in recent years. Suzuki H, Coppo R, and Feehally J took the first place parallelly with 18 articles. Japan contributes the most articles, accounting for 27.3% of all the publications. The institutions with the most publications were Juntendo University and University of Alabama Birmingham. 453 keywords were concluded in the analysis, which mainly focus on the mucosal pathogenesis and therapy of the IgAN. The top five co‐cited reference cluster are “aberrantly glycosylated IgA,” “corticosteroids,” “animal models,” “*o*‐glycosylationm” and “microRNA‐630.” The most recently burst of keyword is “tonsillectomy” and “gut.”

**Conclusion:**

This was the first bibliometric analysis to systematically analyze the mucosal immunity in IgAN, which obtained the current status and indicated the future research hotspots and development trends. The gut microbiota and the related therapy‐targeted mucosal immunity might be the future research hotspot.

## Graphical abstract

This is an original study focused on mucosal immunity in IgA nephropathy. In this study, we retrieved mucosal immunity in the IgA nephropathy field from the Web of Science database and analyzed the literature characteristics and research hotspots using bibliometric analysis tools. This work provides readers with a scientific and comprehensive view of the structure of knowledge in the field of mucosal immunity in IgA nephropathy, providing research hotspots, frontiers, development trends, and a theoretical basis for subsequent scientific exploration.

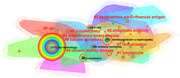

## 1 INTRODUCTION

IgA nephropathy (IgAN) is the most common primary glomerulonephritis all over the world, and about 20% to 40% of patients progressed to end‐stage renal disease in 10–20 years.^1^ IgAN is characterized by the deposition of glycosylation deficient IgA1 in the glomerular mesengium. It always manifests by the macroscopic hematuria simultaneous presented with mucosal infections, such as the infection of the upper respiratory tract, gastrointestinal tract, or urogenital tract.^2^ The realization of the mucosal immunity in IgAN has been raised shortly after the report of the disease.^3^ The mucosal immune system‐kidney axis, such as the tonsil‐kidney axis and the gut‐kidney axis in IgAN, have been the research hotspot for many years,^4^ and researchers have provided evidence supporting mucosal immunity dysregulation in IgAN.^5^

The mucosal immune system contains lymphoid tissues distributed in the oral cavity, skin, respiratory tract, gastrointestinal tract, and urogenital tract.^6^ Nasopharynx‐associated lymphoid tissue (NALT) has been reported to be involved in the pathogenesis and progression of IgAN.^7^, ^8^, ^9^ Dysbiosis of the gut microbiome is thought to be involved in the overproduction of IgA.^10^ On account of the importance of mucosal immunity in IgAN, an increasing number of studies have focused on this aspect, and efforts have been made to explore novel therapies for mucosal immunity to prevent or delay the development of IgAN.

Bibliometric analysis, proposed by Pritchard in 1969, is an effective method to evaluate the overall trends in a given field.^11^ It can be used to analyze basic information, such as publication year, authors, institutions, countries, and citing frequency.^12^ With the help of some software, the research hotspots, the most influential nodal publications, and the research gaps can be quickly understood in the related area to predict the future trends.^13^ CiteSpace, a Java application, is a kind of visualization tool used in bibliometric analysis, which can be applied to explore quantitative information and development trends in a specific research field.^14^

Despite bibliometrics being widely used in the analysis of scientific research, there is no visual quantitative analysis for mucosal immunity in IgAN. In this study, we retrieved the related articles about mucosal immunity in IgAN from the Web of Science database and analyzed the current status and research hotspots from multiple perspectives by the tool CiteSpace. The objective of this study is to provide a more intuitive and comprehensive understanding of the research about mucosal immunity in IgAN and indicate the direction of future studies to some extent.

## 2 MATERIALS AND METHODS

The literature data was collected from the Web of Science Core Collection (WoSCC) on October 17, 2022. The search strategy was: TS = IgA nephropathy AND TS = (“mucosal” OR “tonsil*” OR “gut” OR “intestin*”) AND TS = (“immunology” OR “immunity” OR “immune” OR “immunization”). Only articles and reviews were gathered in our analysis. The time scope was set from 1990 to 2022. XC and ZY retrieved and downloaded the data independently, exported the full records cited references to a plain text file, and stored the data in download_txt format. These records were imported into the CiteSpace 6.1.R3 for visualized analysis.

**Figure 1:**
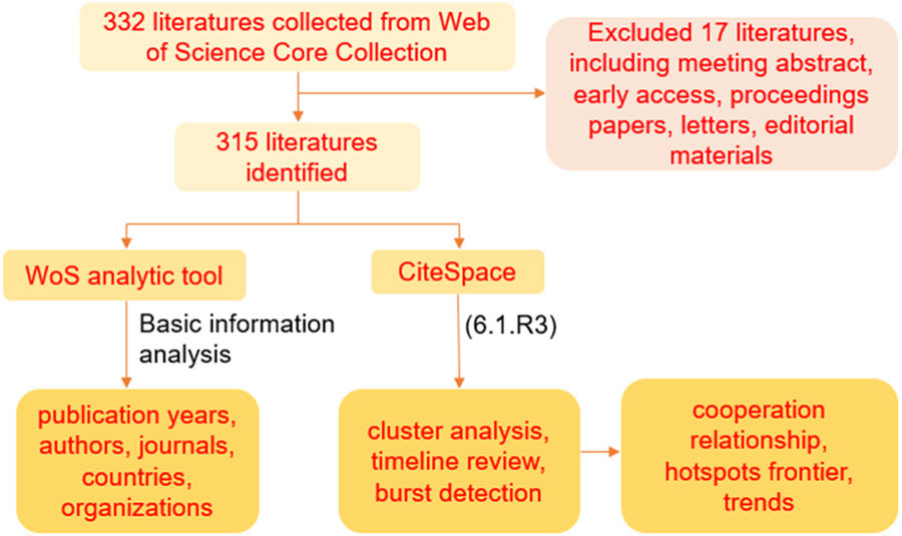
The workflow of bibliometric analysis of mucosal immunity in IgA nephropathy.

Microsoft Excel 2019 was applied to draw the plotting and histogram diagram. CiteSpace 6.1.R3 was employed to conduct a visualization bibliometric analysis of mucosal immunity in IgA nephropathy, including authors, countries, journals, keywords, organizations, references, the bursts of keywords and references, and the timeline of keyword clusters and reference clusters (Figure 1).

## 3 RESULTS AND ANALYSIS

### 3.1 The trend of annual publication quantity

**Figure 2:**
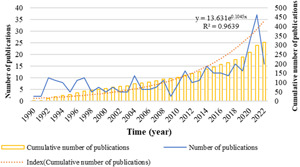
The quantities of annual publications of mucosal immunity in IgA nephropathy.

Using the search strategy outlined above, a total of 315 publications from 1990 to 2022 were included in the analysis. The annual number of mucosal immunity in IgA nephropathy‐related publications fluctuated over time. Increasing numbers of researchers started to investigate this field in 2020 and 2021, which caused a faster increase in the number of publications, and the cumulative number of publications increased gradually (Figure 2). Although studies on mucosal immunity in IgA nephropathy have elevated significantly during recent years, it remains an area that worth exploring further.

### 3.2 Analysis of authors' distribution

More than 530 authors contributed to the 315 literature about mucosal immunity in IgA nephropathy published in WoSCC. Among the top 20 prolific authors, Asians, including Chinese and Japanese, took part of 40%, which is in accordance with the high mobility of IgA nephropathy in Asia, indicating the mucosal immunity in IgA nephropathy is a hot topic among Asian researchers. Suzuki H, Coppo R, and Feehally J take the first place parallelly with 18 articles, followed by Barratt J, Suzuki Y, and Tomino Y with 15 articles separately. The American author Novak J and Brazil author Daha M take seventh and eighth place with 12 and 11 articles, respectively.

### 3.3 Analysis of journals distribution

The articles related to mucosal immunity in IgA nephropathy are distributed among more than 146 journals. Nephrology dialysis transplantation ranks first place with 18 publications in the area of mucosal immunity of IgA nephropathy. It is followed by *Kidney International* (15 publications), *Acta oto Laryngologica* (13 publications), *Clinical and Experimental Immunology* (13 publications), *Nephrology* (12 publications), *Pediatric Nephrology* (11 publications), *Journal of Nephrology* (8 publications), *Nephron* (8 publications), *Seminars in Immunopathology* (7 publications), and *Clinical and Experimental Nephrology* (6 publications). Among the top 10 journals, *Kidney International* has the highest impact factor (IF) of 18.998. Nephrology dialysis transplantation, with the highest number of publications in the field, has an IF of 7.186. Therefore, the vast majority of publications related to mucosal immunity in IgA nephropathy are of high quality and worth further investigation.

### 3.4 Analysis of countries' distribution

Forty‐five countries involved in the publication of literature about mucosal immunity in IgA nephropathy according to the retrieval results of the WoSCC database. Japan contributes the most with 86 publications, accounting for 27.3% of all the publications, followed by United States (61 publications), China (57 publications), England (34 publications), Italy (30 publications), and France (29 publications), which is accordance with the author distribution.

### 3.5 Analysis of organization distribution

According to the WoSCC database, almost 200 organizations are involved in the research of mucosal immunity in IgA nephropathy. Juntendo University and University of Alabama Birmingham took the first place with 21 works of literature separately, followed by University Hospitals of Leicester NHS Trust with 20 publications and Udice French Research University with 19 works of literature. Organizations from Japan and the United States predominate in the field of mucosal immunity in IgA nephropathy.

### 3.6 Cluster analysis of co‐appearance keyword

**Figure 3:**
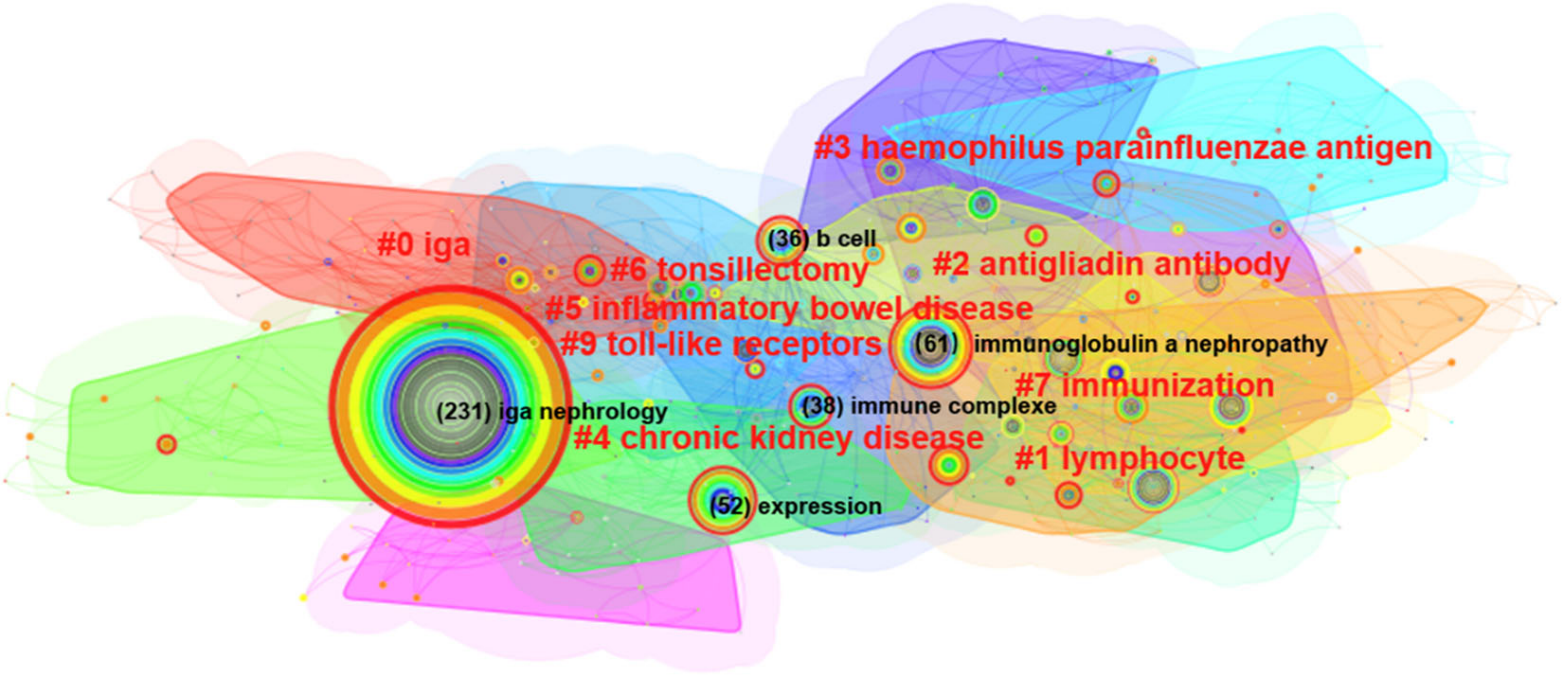
Cluster analysis of keyword co‐appearance of mucosal immunity in IgA nephropathy.

**Figure 4:**
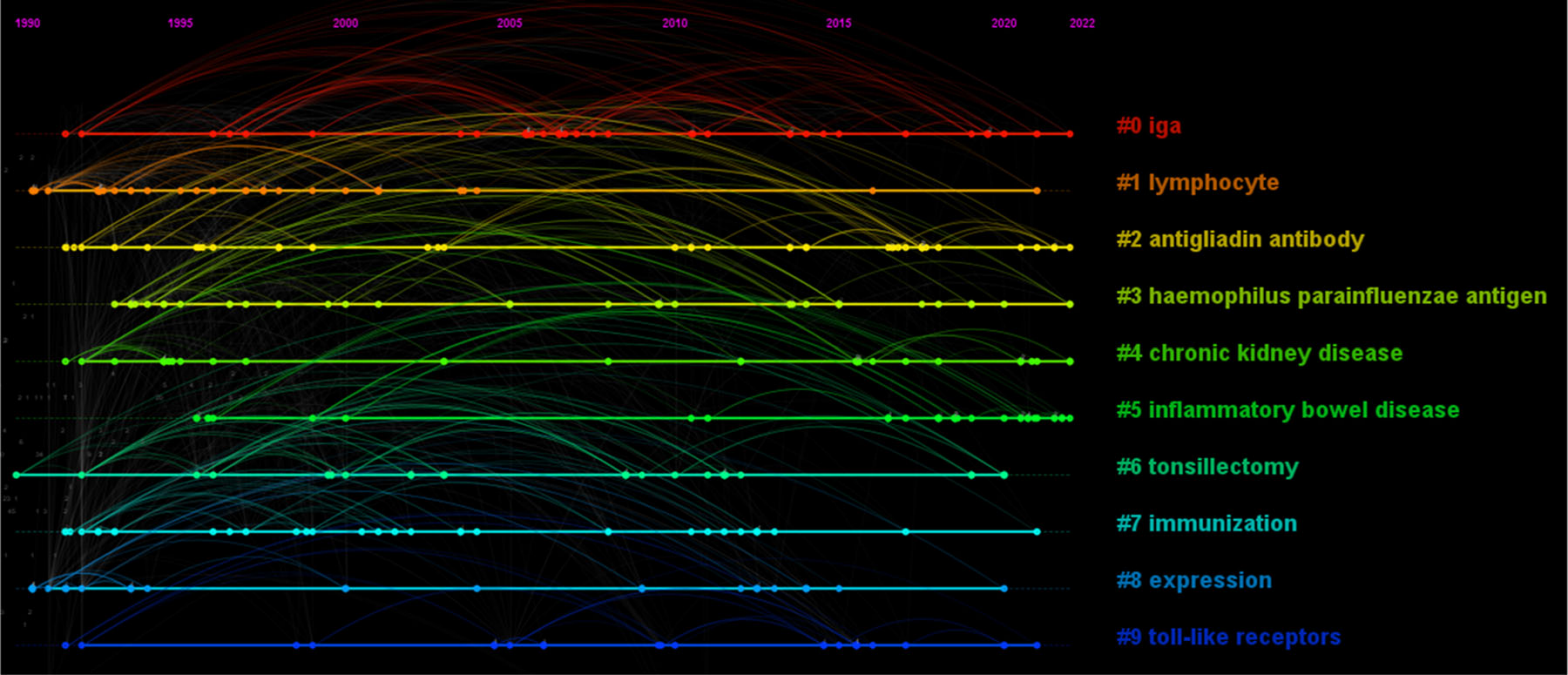
The timeline view of keyword co‐occurrence of mucosal immunity in IgA nephropathy.

Keywords are a series of words that define the key content of your essay. They are the key between what people search for and what you offer to fill that need. Therefore, we can have a general perception of the characteristics and themes of the literature through keyword analysis. There were 453 nodes and 2042 links in the keyword co‐appearance network by CiteSpace 6.1.R3. The top 20 most frequent keywords were shown in Table 1. “IgA nephropathy” was the most usually appeared keyword in the relevant literature, followed by “Immunoglobulin A nephropathy,” “expression,” “glomerulonephritis,” “immune complexe,” “B cell,” “cell,” “antibody,” “tonsillectomy,” “steroid pulse therapy,” “mucosal immunity,” “galactose deficient IgA1,” “*o*‐glycosylation,” and “gut microbiota.” Cluster analysis of the co‐appearance keyword was conducted (Figure 3). Clusters located in the top five are IgA, lymphocyte, antigliadin antibody, hemophilus parainfluenzae antigen, and chronic kidney disease. The keyword co‐occurrence timeline view showed the development and change of keywords in each cluster (Figure 4).

**Table 1:** Top 20 keywords of mucosal immunity in IgA nephropathy.

Ranking	Counts	Centrality	Keywords
1	231	0.39	IgA nephropathy
2	61	0.16	Immunoglobulin A nephropathy
3	52	0.25	Expression
4	39	0.18	Glomerulonephritis
5	38	0.17	Immune complexe
6	36	0.16	B cell
7	32	0.10	Cell
8	31	0.09	Antibody
9	30	0.01	Tonsillectomy
10	28	0.02	Steroid pulse therapy
11	27	0.11	Mucosal immunity
12	22	0.05	Galactose deficient IgA1
13	22	0.04	O glycosylation
14	21	0.04	Gut microbiota
15	20	0.13	T‐cell
16	20	0.06	Children
17	20	0.08	Immunoglobulin A
18	20	0.11	Glycosylation
19	19	0.01	Oxford classification
20	19	0.03	Clinical remission

### 3.7 Cluster analysis of co‐cited reference

Reference co‐citation clustering analysis allows us to visually understand relevant research topics and hotspots. Each cluster represents a research frontier in some way, which can prompt relevant scholars to follow the research hotspots. In this circumstance, clustering analysis and a timeline view of reference co‐citation were completed by CiteSpace to analyze the research trend of mucosal immunity in IgA nephropathy.

**Figure 5:**
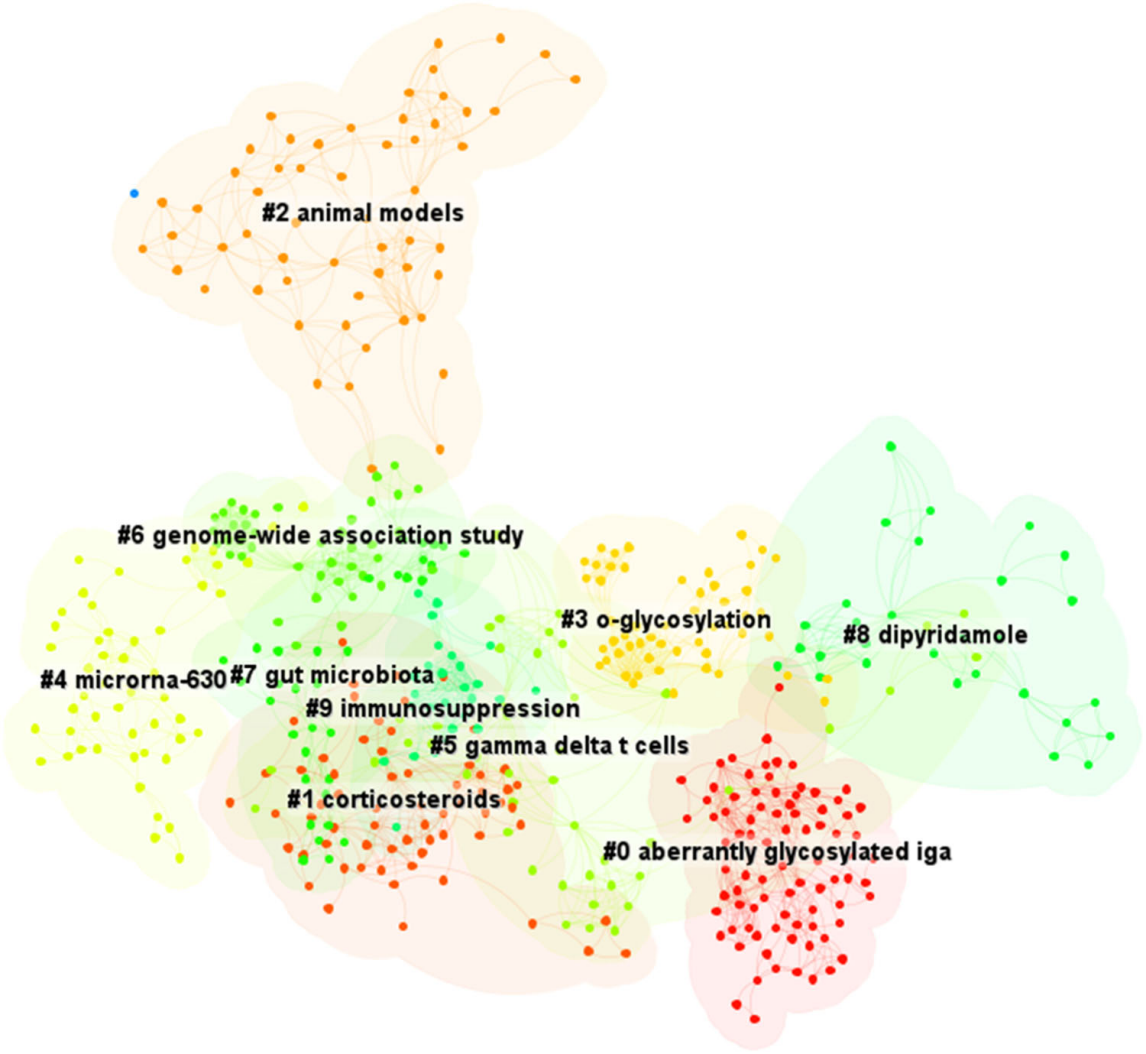
Cluster analysis of reference co‐citation of mucosal immunity in IgA nephropathy.

**Figure 6:**
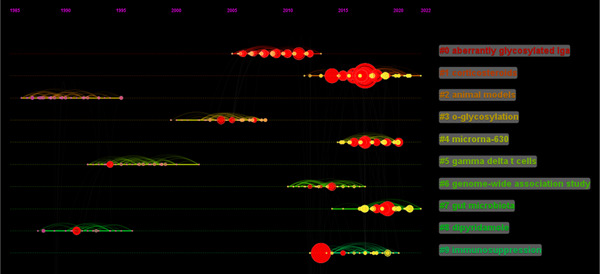
The timeline view of reference co‐citation of mucosal immunity in IgA nephropathy.

A cluster network of reference co‐citation with 801 codes and 2467 links was formed by CiteSpace 6.1.R3. The top 20 most cited references are summarized in Table 2. The most cited literature was published in Lancet by Fellstrom BC in 2017. The result of the reference co‐citation clustering analysis is displayed in Figure 5. Arranged by cluster size, the top five clusters are “aberrantly glycosylated IgA,” “corticosteroids,” “animal models,” “o‐glycosylation,” and “microRNA‐630.” Timeline view of reference co‐citation was shown in Figure 6, which reflected the changes in the research hotspots of the cited literature over time.

**Table 2:** The top 20 most cited references of mucosal immunity in IgA nephropathy.

Ranking	Counts	Centrality	Author	Journal	Year	Vol	Page
1	52	0.14	Fellstrom BC	*Lancet*	2017	389	2117
2	33	0.04	Chemouny JM	*Nephrol. Dial. Transpl*.	2019	34	1135
3	33	0.00	Wyatt RJ	*New Engl. J. Med*.	2013	368	2402
4	29	0.00	Floege J	*Nat. Rev. Nephrol*.	2016	12	147
5	27	0.02	Lv JC	*JAMA*	2017	318	432
6	24	0.00	Muto M	*J. Am. Soc. Nephrol*.	2017	28	1227
7	23	0.00	Kiryluk K	*Nat. Genet*.	2014	46	1187
8	20	0.01	Trimarchi H	*Kidney Int*.	2017	91	1014
9	20	0.02	Watanabe H	*Nephrol. Dial. Transpl*.	2017	32	2072
10	19	0.04	Lafayette RA	*J. Am. Soc. Nephrol*.	2017	28	1306
11	19	0.02	Coppo R	*Semin Nephrol*.	2018	38	504
12	16	0.03	Gharavi AG	*Nat. Genet*.	2011	43	321
13	16	0.01	Suzuki H	*J. Clin. Invest*.	2009	119	1668
14	15	0.01	Coppo R	*Nephrol. Dial. Transpl*.	2015	30	360
15	15	0.07	Rauen T	*New Engl. J. Med*.	2015	373	2225
16	15	0.03	Coppo R	*Pediatr. Nephrol*.	2018	33	53
17	14	0.00	Lechner SM	*J. Am. Soc. Nephrol*.	2016	27	2622
18	14	0.01	Rizk DV	*J. Am. Soc. Nephrol*.	2019	30	2017
19	13	0.00	Papista C	*Kidney Int*.	2015	88	276
20	13	0.07	Mestecky J	*Annu. Rev. Patholmech*.	2013	8	217

### 3.8 Burst detection of keywords and references

**Figure 7:**
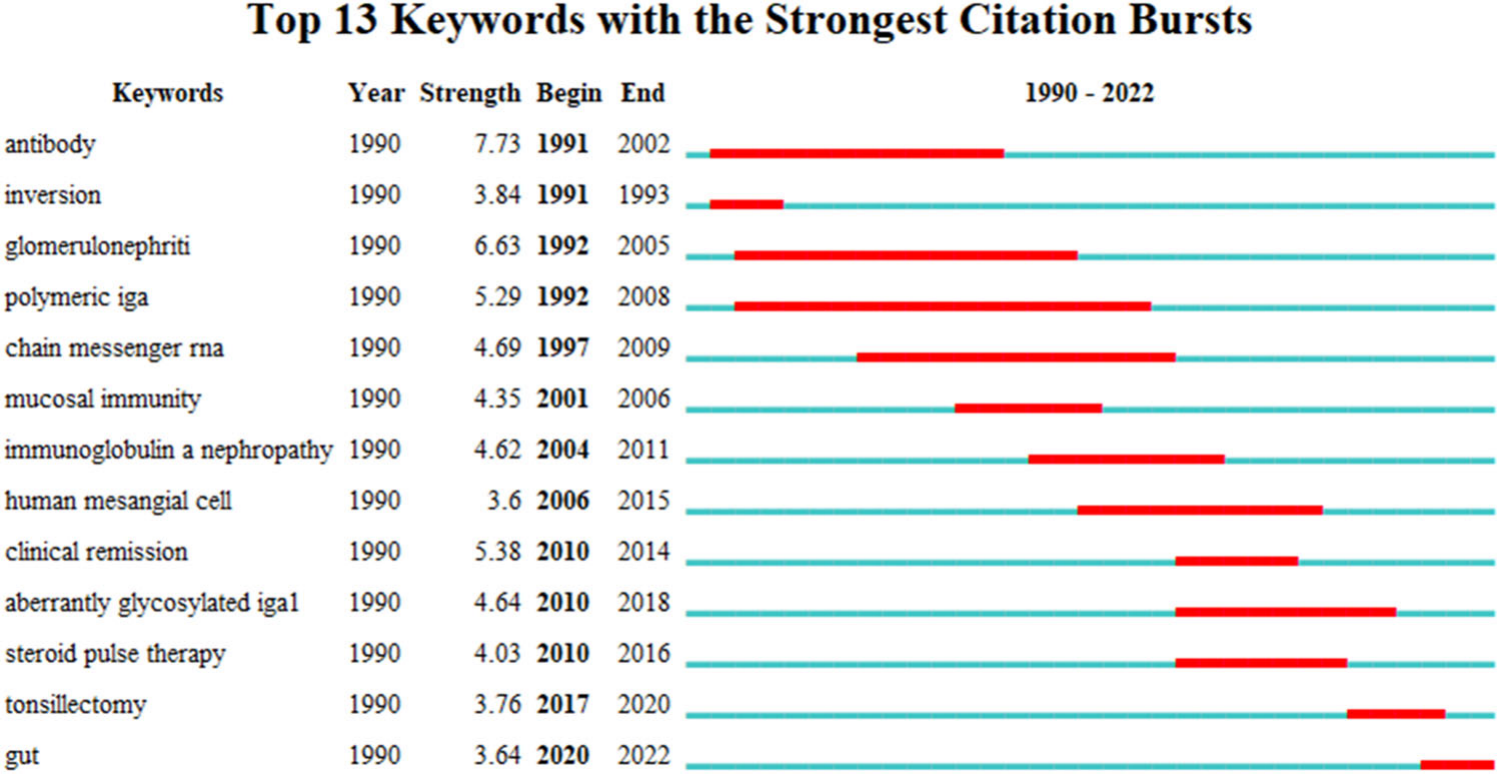
The burst detection of keywords of mucosal immunity in IgA nephropathy.

**Figure 8:**
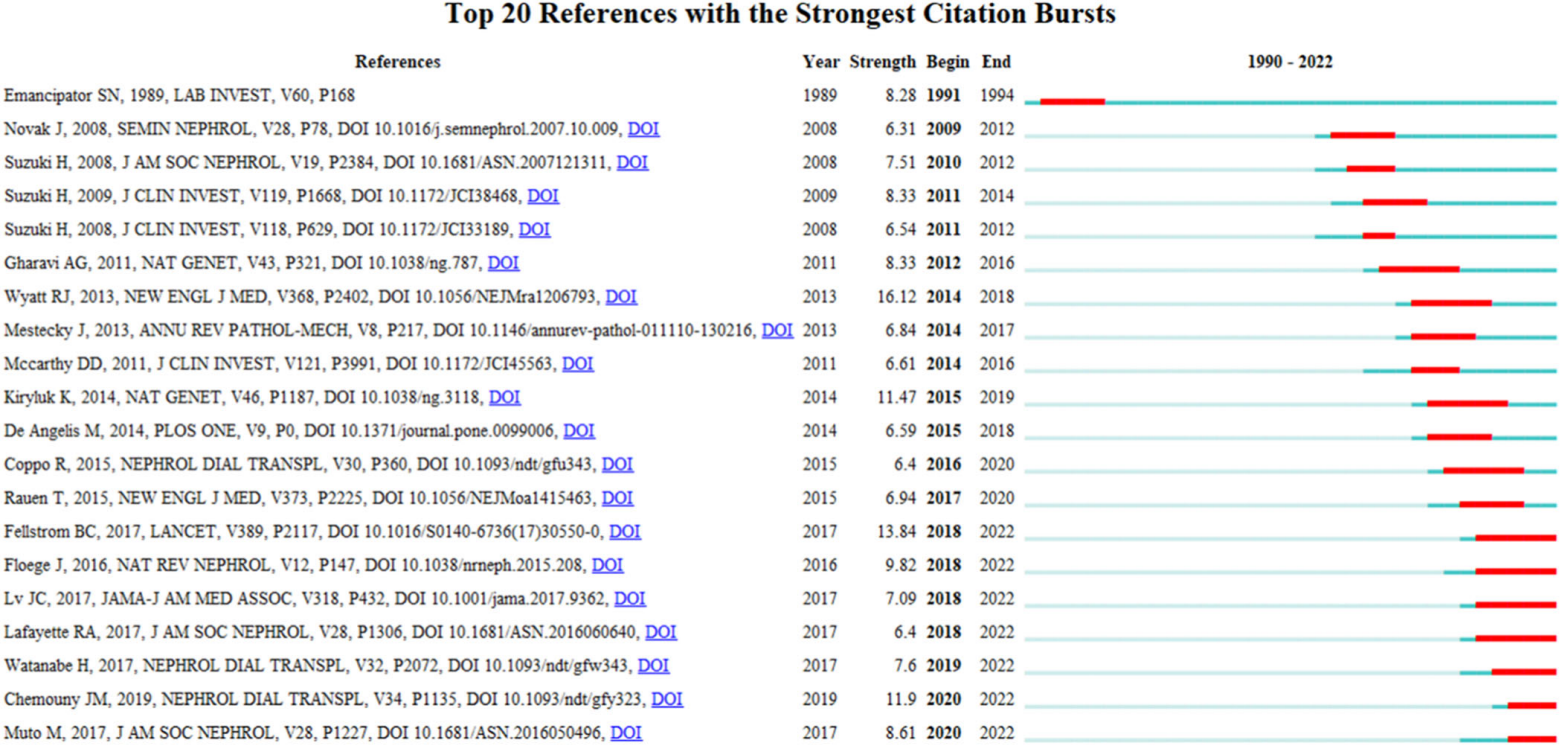
The burst detection of cited references of mucosal immunity in IgA nephropathy.

The function of burst detection in CiteSpace can be used to detect the great changes of the frequency of the keywords or cited literature in a short time. The burst detection of the top 13 keywords was shown in Figure 7, while the top 20 cited references were presented in Figure 8. They are all listed by the burst time. The strongest citation burst of the keyword is “antibody,” while the longest duration burst of the keyword is “polymeric IgA,” and the most recently burst of the keyword is “tonsillectomy” and “gut.” As for the burst of cited references, the strongest citation burst is “Wyatt RJ, 2013,” while the latest citation burst is “Chemouny JM, 2019” and “Muto M, 2017.”

## 4 DISCUSSION

Due to the high incidence of IgA nephropathy and the high rate of progression to uremia, the pathogenesis of IgAN, especially the mucosal immunity, is still a research hotspot in this area. At present, there are much related literature in this field, and we apply bibliometrics to analyze the overall overview of this research domain.

In our study, we performed a literature search about mucosal immunity in IgAN in the Web of Science Core Collection, and a total of 315 publications from 1990 to 2022 were included in the analysis. The number of articles was gradually on the rise, which indicated the importance and potential of the research. The incidence of IgA nephropathy is 30%–60% in Asia, 20%–30% in Europe, and less than 5% in Africa.^15^, ^16^ According to our analysis, Asian countries, mainly Japan and China, have the highest number of publications, which is consistent with the epidemiology of IgA nephropathy.^17^ United States contributes 61 articles, accounting for 19.4% of all the publications. The United States also played a leading role, which might be related to the strong financial support for scientific research in the United States.^18^ At present, Suzuki H, Coppo R, and Feehally J take the first place parallelly with 18 publications in our research aspect, and they come from Juntendo University, University of Turin and University of Leicester, respectively, which is consistent with the incidence of IgAN. Juntendo University, University of Alabama Birmingham, and University Hospitals of Leicester NHS Trust, located in Japan, United States, and England separately, are the top three institutions with the largest number of publications. Japanese universities and scholars have made great contributions in this area, which may be related to the fact that IgAN is more prevalent and more study of tonsillectomy in Japan.^15^ Why the incidence of IgA nephropathy in Japan and China is higher than that in Europe and the United States？Is it caused by the difference in population screening, genetic differences, or differences in diet or climate? As some patients with IgA nephropathy are asymptomatic and do not have regular physical examinations, and this part of patients is the so‐called “fish escaping from the net,” which will lead to a decrease in the calculated incidence, so large‐scale population urine screening needs to be gradually promoted. This could be a research direction to investigate the correlation between regional research output and the percentage of IgAN in a population in the future.

As for the co‐appearance keywords through Citespace, “mucosal immunity,” “galactose deficient IgA1,” “*o*‐glycosylation” and “gut microbiota” are the hot words that also hint the research hotspots in the pathogenesis of IgAN, which is consistent with the “four‐fit” process of the IgAN pathogenesis. The mucosal immune system is reported to be a key factor in triggering IgAN, and the “gut‐kidney axis” is proposed to be involved in its development.^19^ Gut microbiota dysbiosis was reported in the pathogenesis of IgAN, in which *Paraprevotella* and *Streptococcus* showed a higher proportion in patients with IgAN,^20^ and the disturbance of intestinal microflora may be associated with the severity of IgAN.^21^ Ai et al.^22^ have reported recently that there is a specific emphasis on the present state of intercommunication between gut microbiota and kidney diseases. Wang et al.^23^ have also demonstrated that current research hotspots are mainly multicenter studies related to IgA nephropathy and its exploration with gut microbiota. IgA is the predominant Ig class in the gut and has a negative correlation with *Clostridium ramosum*, *Eggerthella lenta*, *Lactobacillus casei*, and *Leuconostoc mesenteroides*.^24^ Xie et al.^25^ recently reported that chimeric fusion between *C. ramosum* IgA protease and IgG Fc can clear the IgA deposits in IgAN mouse models.

“Aberrantly glycosylated IgA,” “corticosteroids,” “animal models,” “*o*‐glycosylation,” and “microRNA‐630” are the top five reference co‐citation clusters. Aberrantly glycosylated IgA and *O*‐glycosylation are still the research hotspots in the pathogenesis of IgAN.^26^, ^27^ The level of IgA1 *o‐*glycan sialylation was positive associated with eGFR.^27^ As the effect of glucocorticoids in IgAN is still uncertain, many studies were designed to evaluate the efficacy and adverse effects of corticosteroids in IgAN, among which the most valuable one is the TESTING randomized clinical trial. Oral methylprednisolone in IgAN at high risk of progression for 6–9 months can reduce the risk of kidney function decline, kidney failure, and death caused by kidney disease.^28^ Therefore, it was of great importance to study mucosal immunity and therapy in IgAN in depth.

The most cited literature concerning the phase 2b NEFIGAN trail about targeted‐release budesonide (Nefecon) in IgAN, was published in Lancet by Fellstrom BC in 2017,^29^ which suggested that high‐quality articles are of common concern and can provide more cutting‐edge information to relevant researchers. Nefecon is a kind of oral glucocorticoid budesonide that can deploy in the ileum and deliver the glucocorticoid locally.^30^ The Phase 3 NefigArd trial tested the efficacy and safety of 9 months of treatment with budesonide and showed clinically important improvements in UPCR and eGFR.^31^ Although the treatment‐targeted mucosal immunity has become a research hotspot and has achieved initial results, further studies are needed to be implemented.

The burst detection was used to detect the great changes in frequency of the keywords or cited literature in a short time. In our analysis, the most recently burst of keywords is “tonsillectomy” and “gut.” No matter tonsil or gut all belong to mucosal immunity.^32^ Tonsillectomy in IgAN patients has always been controversial, for example, tonsillectomy was not recommended to be performed as a treatment for IgAN in Caucasian patients, however, it was still suggested in some countries for the treatment of recurrent tonsillitis‐related IgAN patients, especially in Japan and China.^33^, ^34^, ^35^ The reasons for the racial differences in the therapeutic effect of tonsillectomy warrant further investigation.

The ultimate goal of continuous in‐depth research on the pathogenesis of IgA nephropathy is to select the optimal treatment. At present, the 2021 KDIGO Clinical Practice Guideline recommends the use of 6‐month corticosteroids for patients with IgA nephropathy who are at high risk of disease progression.^33^ However, there is still controversy due to the significant side effects of corticosteroids. At present, new treatments for IgAN are gradually emerging, such as Nefecon, B cell proliferation and differentiation inhibitors, as well as blockade of complement components. The emergence of new therapies is also accompanied by new issues, such as the selection of treatment timing and whether there are biomarkers to guide treatment selection. Further research may be needed to explore relevant issues in the future.

This is the first bibliometric analysis to systematically analyze the mucosal immunity in IgAN, which demonstrates detailed content and a better insight into research focuses and trends for researchers in this field. However, we still have several limitations. First, the publications were all retrieved from the WoSCC, which may result in missing data from other databases. Second, only English articles were involved in our study, resulting in the non‐English literature being excluded. Third, we manually filtered the raw records that were less relevant to the study, but this may have a selection bias.

## 5 CONCLUSION

In summary, we systematically assessed the relationship of mucosal immunity in IgAN based on the bibliometric analysis. The number of publications increased in recent years, which indicated many scholars were interested in this aera. We thoroughly reviewed the authors, journals, institutions, countries, co‐appearance keywords, and co‐cited references of the publications in this field, indicating the future research hotspots and development trends. The future research hotspot might be the gut microbiota and the related therapy‐targeted mucosal immunity. Scholars specialized in this field can get the research hotspots and frontiers in mucosal immunity of IgAN from this study.

## AUTHOR CONTRIBUTIONS

**Xian Chen**: Data curation; funding acquisition; software; writing—original draft. **Zhe Yan**: Data curation. **Qing Pan**: Data curation. **Chunxia Zhang**: Data curation. **Yakun Chen**: Investigation; supervision. **Xuzhi Liang**: Data curation. **Shaomei Li**: Supervision. **Gang Wu**: Funding acquisition; writing—review & editing.

## CONFLICT OF INTEREST STATEMENT

The authors declare no conflict of interest.

## Data Availability

All data generated or analyzed during this study are included in this published article, and are available from the corresponding author on reasonable request. The data that support the findings of this study are available from the corresponding author upon reasonable request.
